# A Simplified and Efficient Protocol for DNA Isolation from Deer Antlers and Prepared Trophy Skulls

**DOI:** 10.3390/ani16071056

**Published:** 2026-03-30

**Authors:** Eszter Lőrincz, Lajos Molnár, Norbert Bleier, Miklós Marosán, Zsombor Wagenhoffer, Orsolya K. Zorkóczy, Petra Zenke

**Affiliations:** 1 Department of Animal Breeding and Genetics, Institute for Animal Breeding, Nutrition and Laboratory Animal Science, University of Veterinary Medicine Budapest, Istvan u. 2, H-1078 Budapest, Hungarywagenhoffer.zsombor@univet.hu (Z.W.); zorkoczy.orsolya.krisztina@univet.hu (O.K.Z.); 2 Department of Genetics, Hungarian Institute for Forensic Sciences, Mosonyi u. 9, H-1087 Budapest, Hungary; 3 Pásztó District Office of the Nógrád County Government Office, Kölcsey u. 35, H-3060 Paszto, Hungary; molnar.lajos3@nograd.gov.hu; 4 Wildlife Management Department, Ministry of Agriculture, Kossuth Lajos tér 11, H-1055 Budapest, Hungary; norbert.bleier@am.gov.hu; 5 Department of Exotic Animal-, Wildlife-, Fish- and Honeybee Medicine, University of Veterinary Medicine Budapest, Istvan u. 2, H-1078 Budapest, Hungary; marosan.miklos@univet.hu; 6 Department of Water Management and Natural Ecosystems, Albert Kázmér Faculty of Agricultural and Food Sciences, Széchenyi István University, Vár Square 2, H-9200 Mosonmagyaróvár, Hungary

**Keywords:** DNA extraction, antler samples, trophy skulls, deer species, microsatellite genotyping, phenol-chloroform purification, wildlife forensics, conservation genetics

## Abstract

A simple, fast, and cost-effective organic solvent-based protocol was developed for DNA extraction from deer antlers and prepared trophy skulls, eliminating the need for commercial kits or cryogenic grinding. The method combines bead-based mechanical homogenization with a 4 h enzymatic digestion in EDTA buffer containing N-lauryl sarcosine and Proteinase K, followed by phenol–chloroform–isoamyl alcohol purification and centrifugal filtration. DNA quality and quantity were evaluated using agarose gel electrophoresis, Qubit fluorometry, and Nanodrop spectrophotometry. The protocol was tested on 60 samples, comprising 30 antlers and 30 pedicle parts from prepared trophy skulls of roe deer (*Capreolus capreolus*), fallow deer (*Dama dama*), and red deer (*Cervus elaphus*). To assess suitability for downstream applications, species-specific microsatellite markers were amplified using multiplex PCR, successfully generating complete genotypes from all 60 samples. These results, along with a demonstrated case study, confirm that the developed protocol provides high-quality DNA suitable for molecular genetic investigations, enabling reliable genotyping from small amounts of both antler and processed trophy materials in forensic and conservation contexts.

## Simple Summary

This study developed a simple and affordable method for extracting DNA from deer antlers and trophy skulls without needing expensive kits or equipment. We focused on three common European deer species—red deer, roe deer, and fallow deer—and tested 60 samples taken from prepared trophy skulls with antlers. After parallel pre-screening with two methods, a modified organic process was applied to isolate DNA suitable for genetic analyses. The quality of the DNA was confirmed using several laboratory techniques, and we demonstrated that this method works across different deer species and sample types obtained from trophies. This approach avoids the need for specialized tools such as liquid nitrogen or automated extraction platforms while still producing high-quality DNA, even from hard materials like prepared trophy skulls. Importantly, this method was successfully applied in a real-world case, confirming its usefulness for wildlife monitoring, hunting law enforcement, and research. This work is valuable because it makes advanced genetic testing more accessible and practical for field researchers, conservationists, and forensic investigators.

## 1. Introduction

Antlers are developed in most species of deer, typically only males, and are composed of rapidly growing solid tissue that initially forms from cartilage and later transforms into bone [1]. Antlers are not permanent structures; they regrow every year, and their size and shape reflect the animal’s age, health, and genetic fitness.

Analysis of antler samples holds significant potential across various fields, including conservation, forensics, biomedicine, the ecological and evolutionary dynamics of deer populations, and prehistoric and ancient DNA research [2]. With advancements in DNA extraction techniques, the study of antlers has proven valuable for understanding the genetic structure of these species (Table S1). Early studies focused on mitochondrial DNA from Giant deer (*Megaloceros giganteus*) specimens, demonstrating the utility of antlers in recovering historical genetic data and contributing to phylogenetic analysis [3]. Other research has proven that it is possible to isolate nuclear DNA microsatellites from old, museum-preserved antlers, enabling multi-generational studies [4,5]. Lopez and Beier (2012) further showed that naturally shed and weathered antlers can retain usable DNA over time, making them valuable for both historical and contemporary population studies [6]. Antlers also play a role in forensic genetics, particularly in individual identification, allowing for comparisons with registered trophies and detection of illegal hunting activities [7]. In biomedical research, antlers serve as models for rapid mammalian growth and cartilage formation, with studies highlighting the role of epigenetic regulation in cartilage differentiation [8]. Research on sika deer (*Cervus nippon*) has examined the role of the osteopontin gene in tissue development, while investigations into velvet antler growth underscore its biomedical and economic significance [8,9,10]. In traditional Asian medicine, deer products—such as antlers, meat, skin, and bones—remain highly valued. However, the high market demand has led to counterfeit products, often mixed with tissues from other animals (e.g., pigs, cows, sheep). DNA barcoding has proven effective in verifying the species origin of these products, including antlers [11,12]. Furthermore, for RNA-based transcriptomic studies on rapid growth and annual regeneration, antler velvet and mesenchyme tissues provide reliable sources [13,14].

**Figure 1:**
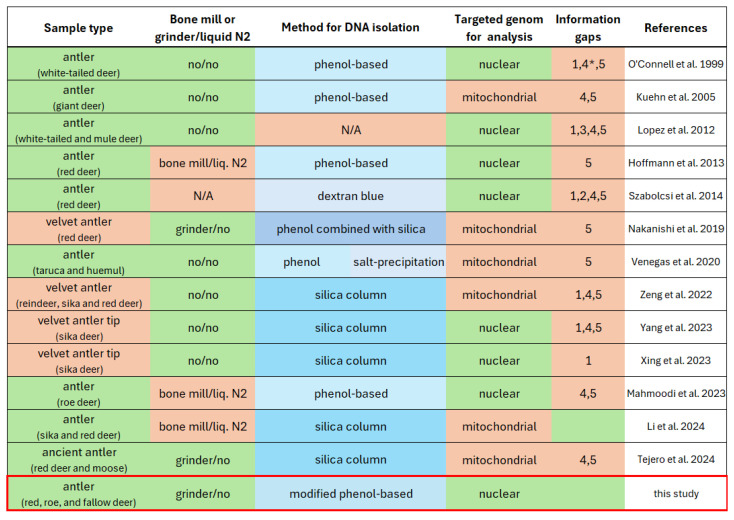
Overview of published studies on DNA extraction from antlers, including sample type, use of bone mill or liquid nitrogen, extraction method, targeted downstream analysis, and information gaps: (1) sample weight, (2) use of grinding or liquid nitrogen, (3) DNA isolation protocol, (4) extracted DNA yield, and (5) DNA purity based on A260/280 ratios. N/A: no data available. Background colors indicate methodological assessment: red denotes aspects to be avoided, green indicates suitable conditions, and blue shades differentiate groups of extraction approaches for clarity. Most previously published studies primarily focused on mitochondrial DNA (mtDNA) analysis, whereas the present study specifically evaluates nuclear DNA recovery, which requires higher DNA quality and integrity for downstream applications. Our proposed method, planned for future implementation, is highlighted with a red-bordered row. References: O’Connell et al. 1999 [5], Kuehn et al. 2005 [3], Lopez et al. 2012 [6], Hoffmann et al. 2013 [4], Szabolcsi et al. 2014 [7], Nakanishi et al. 2019 [12], Venegas et al. 2020 [16], Zeng et al. 2022 [17], Yang et al. 2023 [10], Xing et al. 2023 [8], Mahmoodi et al. 2023 [18], Li et al. 2024 [11], Tejero et al. 2024 [2]. 4*: DNA yield assessed by agarose gel electrophoresis only.

DNA extraction and purification are essential steps in various scientific disciplines, and the primary goal is to obtain pure, high-quality DNA suitable for downstream applications such as PCR, microarray technologies, and sequencing. Studies involving bone tissue—including antlers—have shown that using liquid nitrogen in combination with bone mills can improve DNA yield by preserving DNA integrity [15]. Successful extraction and subsequent short tandem repeat (STR) genotyping or mitochondrial gene sequencing from red deer (*Cervus elaphus*) and sika deer antlers have typically relied on this approach [4,11]. However, these tools are expensive and often unavailable in basic laboratory settings. For more accessible and preservation-friendly sampling, Venegas et al. (2020) compared multiple DNA extraction techniques for antler tissue [16]. While all methods were suitable for mitochondrial gene sequencing, they required a large quantity (10 g) of connective tissue. Most studies used either phenol-based extraction or silica column-based methods. The latter was favored for fresher samples, such as velvet antlers or antler tips [8,10,17], and sometimes used in combination with phenol extraction [9] (Table 1 and Table S1). Although silica column-based techniques are non-toxic and commonly used, they generally produce high yields only when (1) large amounts of fresh, non-ossified tissue are available, and (2) a bone mill is used. While the exact extraction protocol is available in most of these publications, there is a lack of data on the quantity and quality of the extracted DNA. The figure below clearly illustrates that, based on the current literature data [2,3,4,5,6,7,8,9,10,11,12,14,16,17,18], there is no detailed protocol available that ensures the extraction of sufficient quantity and quality from antler samples without a bone mill, in a reproducible manner and supported by various measurements, for a wide variety of genetic research targeting the nuclear genome (Figure 1).

This study aimed to develop an optimized phenol-based DNA extraction protocol for antlers and antler pedicles obtained from prepared trophy skulls. Building on a preliminary comparison of silica column-based and organic extraction methods, our objective was to establish a workflow that eliminates the need for bone milling, liquid nitrogen, or prolonged decalcification, while still ensuring adequate DNA yield, purity, and integrity. We further aimed to assess DNA quality using multiple quantitative and qualitative metrics and to evaluate its suitability for genotyping with microsatellite multiplexes, including panels partially developed in the course of this work. A comparative assessment was performed across three ecologically and forensically important deer species—roe deer (*Capreolus capreolus*), fallow deer (*Dama dama*), and red deer (*Cervus elaphus*)—noting that no antler-derived genetic data currently exist for fallow deer. Finally, we aimed to test the applicability of the protocol on antler and antler pedicle in a real investigation involving suspected violations of hunting regulations.

In early 2025, a red deer stag was mistakenly harvested in the Transdanubian region of Hungary after having recently shed its antlers. Under Hungarian hunting regulations, antlerless stags are not permitted to be taken. Believing the animal to be a female, which was legally huntable at the time, the hunter shot the deer. Upon discovery, the animal was identified as a male, resulting in sanctions by the Hungarian hunting authority.

As per national practice, the hunting fee is calculated retrospectively based on the trophy weight (skull and antlers). Since no antlers were present, the local hunting association imposed the legally defined game management fee for an antlerless stag (approximately €2.500). The hunter subsequently located one of the shed antlers near the hunting site and requested a genetic analysis to confirm if the antler and skull belonged to the same individual. If confirmed, the estimated full trophy weight could be used to calculate the fee based on standard trophy pricing, potentially greatly reducing the amount owed under game management regulations.

## 2. Materials and Methods

### 2.1. Sample Collection

**Figure 2:**
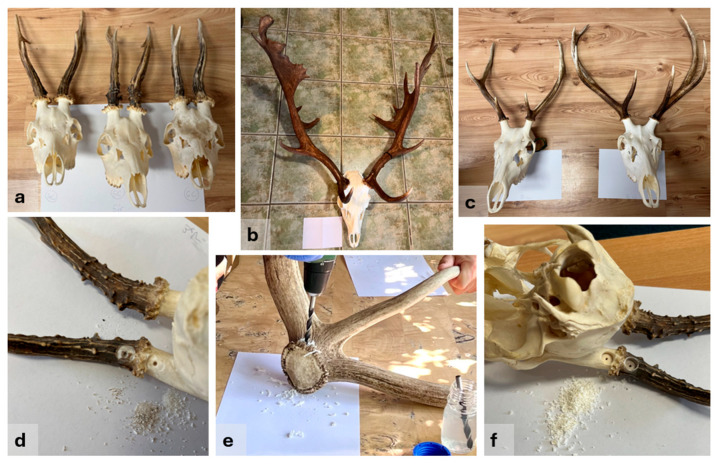
Prepared trophies investigated from three cervid species and the sampling process: (**a**) roe deer (*Capreolus capreolus*) skulls with antlers; (**b**) fallow deer (*Dama dama*) skull with antlers; (**c**) red deer (*Cervus elaphus*) skulls with antlers; (**d**) drilling of the top layer on the antler base and pedicle; (**e**) drilling on the antler base; (**f**) drilling sites on the pedicle and antler base, with corresponding shavings from the pedicle.

**Figure 3:**
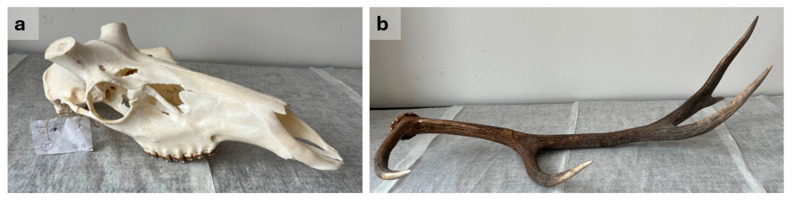
Case type samples: (**a**) prepared deer skull and (**b**) red deer (*Cervus elaphus*) antler—about 1 m long—found in the forest.

Trophies were harvested within the last 30 years, originating from 10 roe deer (*Capreolus capreolus*), 10 red deer (*Cervus elaphus*), and 10 fallow deer (*Dama dama*). Two samples were taken from each trophy, one from the antler and one from the pedicle part, so a total of *n* = 60 samples were tested (Table 1). Trophies (skull with antlers, Figure 2a–c), prepared by boiling in water and treated with a 3% hydrogen peroxide solution, were obtained from private hunters and hunting associations. In addition, two case-type samples—a prepared red deer trophy skull and an antler beam—were provided for genetic analysis by an official judicial expert in wildlife damage (Figure 3). The preliminary hypothesis was that the antler may have originated from a slaughtered red deer stag, necessitating a comparative genetic study.

To remove surface contamination, the sampling area was first abraded with emery paper. After drilling through the surface, the top layer of the antler was discarded (Figure 2d). Shavings were then collected from both the antler base (just above the burr) and the antler pedicle using a sterile 8 mm drill bit (Figure 2e,f). To avoid visible damage, drilling was carried out on the backside of the trophy, using a low rotational speed to minimize heat generation and thereby preserve DNA integrity. The drill penetrated approximately 0.5 cm deep, avoiding the deeper bone marrow. Between each sampling, the drill bit was disinfected with BIB forte eco (Alpro Medical GmbH, Rheinbach, Germany), an instrument disinfectant that provides effective decontamination without causing corrosion.

### 2.2. DNA Extraction and Quantification

As a preliminary comparative study, paired antler and pedicle samples were collected from four individuals per species (8 samples per species in total). DNA was isolated using both a silica column-based method (Qiagen DNeasy Blood & Tissue Kit, Qiagen GmbH, Hilden, Germany) and an organic (phenol-based) extraction method.

The shavings were homogenized using a TissueLyser LT (Qiagen GmbH, Hilden, Germany), a bead-based mechanical disruptor. To minimize heat buildup, the tube adapter was pre-cooled in a freezer for approximately 15 min before use and then loaded with 2 mL U-bottom Eppendorf tubes, each containing 0.1 g of antler shavings and a 5 mm stainless steel bead. The instrument was set to 50 Hz for 1.5 min to achieve a fine powder from the antler shavings. After removing the beads, powdered antler and pedicle materials were decalcified and digested.

In the case of the silica column-based method, we applied a simplified adaptation of the Qiagen protocol “*Purification of total DNA from compact animal bone*“ for the extraction and purification steps (Qiagen, 2020). The extended steps described in the user-developed protocol, such as prolonged EDTA decalcification and bone milling, were not used. Instead, a shortened DNeasy Blood & Tissue Kit workflow was implemented for comparison. In the phenol-based method, the extraction buffer consisted of 600 µL of 0.5 M EDTA (pH 8), 60 µL of 0.5% N-lauryl sarcosine, and 20 µL of Proteinase K solution (PCR grade, 20 mg/mL; ThermoFisher Scientific, Waltham, MA, USA). The tubes were incubated in a thermo-shaker at 50 °C and 900 rpm (revolutions per minute) for two hours, followed by an additional two hours at 56 °C and 900 rpm with an additional 20 µL of Proteinase K solution (20 mg/mL). The extracted DNA was then purified and concentrated using a modified organic extraction followed by dialysis, with the procedure as follows: Briefly, 600 µL of Ultrapure™ phenol–chloroform– isoamyl alcohol (ThermoFisher Scientific, Waltham, MA, USA) was added to the digested solution. After vortexing for 30 s and centrifuging for 5 min at 13,000 rpm, the supernatant was transferred to a sterile 2 mL Eppendorf tube, and the extraction process was repeated. Microcon^®^-100 centrifugal filter units (Merck Millipore, Merck KGaA, Darmstadt, Germany) were used for purification and concentration of the extract. Filter units were pre-moistened with 100 µL Tris-EDTA (TE) buffer. The washing process was performed with TE buffer and centrifuged at 5000 rpm for 20 min until the wash buffer had completely passed through the filter. This process was repeated three times. Finally, purified DNA was recovered in 40 µL of TE buffer, and its quality was assessed on agarose gel using GelRed™ Nucleic Acid Gel Stain (Biotium, Inc., Fremont, CA, USA).

The DNA concentration of each sample was measured using two methods: a Qubit 2.0 Fluorometer (Life Technologies Corporation, Carlsbad, CA, USA) with the dsDNA HS and BR Assay Kits (ThermoFisher Scientific, Waltham, MA, USA), and a Nanodrop OneC Microvolume UV-Vis Spectrophotometer (ThermoFisher Scientific, Waltham, MA, USA). DNA isolates were also tested using the Nanodrop assay for protein purity (A260/280) and organic compound-related purity (A260/230). For high-purity double-stranded DNA, absorbance ratios of A_(260)_/A_(280)_ ≈ 1.8 and A_(260)_/A_(230)_ ≈ 2.0–2.2 are widely accepted as indicators of minimal contamination [19], and the results were statistically analyzed.

### 2.3. Statistical Analysis

All statistical analyses were performed in R v4.5.1 [20]. For the method comparison, paired silica column–phenol-based measurements were available for 24 samples. For each analytical metric (Qubit and Nanodrop concentrations, A_260_/A_280_ and A_260_/A_230_ ratios), paired differences were calculated, explored descriptively, and screened for normality using the Shapiro–Wilk test. Because purity differences were strongly non-normal (A_260_/A_280_: W = 0.881, *p* = 0.0087; A_260_/A_230_: W = 0.396, *p* = 6.3 × 10^−9^), extraction methods were compared using Wilcoxon signed-rank tests; for concentration variables, both paired *t*-tests and Wilcoxon tests are reported.

For the full dataset (60 phenol-extracted samples), DNA concentration variables showed pronounced non-normality and were analyzed using GLMMs with a Gamma distribution and log link (glmmTMB) [21], with species and tissue origin as fixed effects and animal ID as a random effect. Purity ratios were analyzed using LMMs (lme4), with species and tissue origin as fixed factors; the random effect (animal ID) was retained only for A_260_/A_280_, as its inclusion caused singularity in A_260_/A_230_ models. Model selection relied on likelihood ratio tests, and significant terms are reported with χ^2^ statistics, *p*-values, and regression coefficients ± SE. Pairwise contrasts were obtained from estimated marginal means with Holm correction (emmeans) [22].

Agreement between Qubit and Nanodrop measures was assessed using Spearman rank correlations across all samples and within tissue types (antler vs. pedicle).

### 2.4. STR Genotyping

The quality of DNA isolates achieved by the modified phenol-based method was verified using microsatellite-based genetic profiling (*n* = 60). For red deer genotyping with the *DeerPlex I* and *DeerPlex II* multiplexes [7], a reduced reaction volume of 10 μL (instead of 25 μL) was used, while the primers and PCR settings remained unchanged. For roe deer, the *STRoe deer* plex was applied in a 15 μL reaction volume (instead of 20 μL), with all other settings following the original description [23]. For fallow deer DNA samples, a newly developed 8-plex system (Table S2) was applied based on the findings of Zorkóczy et al. [24]. PCR conditions were optimized for fallow deer in a 15 μL reaction volume, containing 4 μL of DreamTaq™ Green DNA Polymerase (ThermoFisher Scientific, Bioscience, Budapest, Hungary), 6 μL of primer mix, 2 ng of DNA template, and PCR-grade H_2_O to reach the final volume. Each polymerase chain reaction (PCR) was performed on 2400 Thermal Cyclers (Applied Biosystems, Life Technologies Corp., Budapest, Hungary) using the following conditions for fallow deer: an initial step at 95 °C for 30 s, followed by 32 cycles of 30 s at 94 °C, 60 s at 59 °C for annealing, and 40 s at 72 °C, with a final extension of 2 min at 72 °C. Negative (no-template) controls were included in each step to monitor contamination.

PCR products from each multiplex system were first checked on a 2% agarose gel and then separated and analyzed by capillary electrophoresis using an ABI Prism 3130XL Genetic Analyzer with the GeneScan-500 LIZ Size Standard (ThermoFisher Scientific, Bioscience, Budapest, Hungary). During fragment analysis using GeneMapper^®^ ID-X software version 1.4, the minimum detection threshold was set at 150 relative fluorescence units (RFU).

To assess the evidential weight of the genetic match regarding the case study, we calculated a likelihood ratio (LR) following the approach outlined by the National Research Council (1996) [25]. Genotype frequencies were derived from the Hungarian allele frequency data [7,26].

## 3. Results

The sampling procedure yielded approximately 0.6 g of antler shavings, sufficient for multiple DNA preparation batches. A total of 84 DNA extractions were performed from antlers and antler pedicles: 2 × 24 parallel isolations for a preliminary comparative study of two extraction methods tested, and 36 additional samples for the validation of the modified phenol-based method (Table 1).

**Figure 4:**
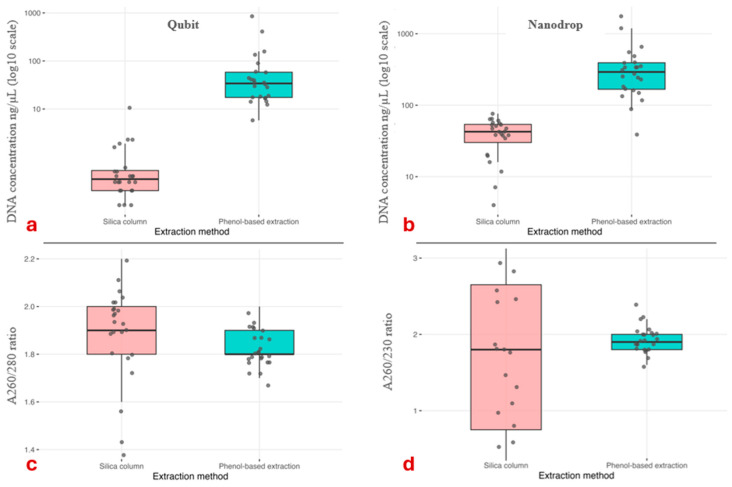
Qubit and Nanodrop DNA concentration (**a**,**b**) and purity (**c**,**d**) measurements by extraction methods. Boxes indicate the interquartile ranges (IQRs), horizontal lines show medians, whiskers extend to 1.5 × IQR, and points represent individual measurements. DNA concentration measured with Qubit (**a**) and Nanodrop (**b**) was significantly influenced by the extraction method; A260/280 (**c**) and A260/230 (**d**) purity ratios measured with Nanodrop did not differ significantly between extraction methods (*p* > 0.1).

In the preliminary comparative study, the phenol-based extraction consistently yielded much higher DNA concentrations (ng/µL) than the silica column protocol in all three cervid species (*n* = 24 paired samples per method; Table 1). The fold increase ranged from approximately 23-fold in roe deer to 40-fold in fallow deer and exceeded 200-fold in red deer using the Qubit measurement, and Nanodrop-based DNA concentrations (ng/µL) showed the same trend, with 5.5- to 16.0-fold higher mean values for the phenol-based protocol across species (Figure S2). In contrast, purity ratios (A260/280 and A260/230) did not differ significantly between extraction methods in any species (all *p* > 0.1), as paired comparisons revealed highly similar values for silica column- and phenol-based DNA extracts, typically falling within the expected range (~1.7–2.0 in the case of A260/280) (Figure 4 and Figure S3).

For the validation study, the following results were obtained from 24 initial samples and supplemented with an additional 36 samples, yielding a total of 60 DNA samples isolated using our modified phenol protocol. Table 1 highlights a notable discrepancy between the two DNA quantification methods. Qubit measurements indicated average concentrations of 44 ng/µL, 174 ng/µL, and 26 ng/µL for roe deer, fallow deer, and red deer samples, respectively, whereas Nanodrop values were substantially higher at 602 ng/µL, 281 ng/µL, and 285 ng/µL. Accordingly, for all 60 samples, Qubit and Nanodrop quantification confirmed that the DNA isolates met the required quality and yield standards. Agarose gel electrophoresis further demonstrated that the extracted DNA was highly intact and contained substantial endogenous content (Table 1; Figure S1). Comparative statistical analyses for species, sample types, and measurement methods are provided in Supplementary Figure S4.

Purity values for both protein-related and organic compound-related metrics showed normal distributions despite generally low absolute values (Table 1). Protein-related purity (A260/280) ranged from 1.6 to 2.0, with a mean of 1.8 across all three cervid species. Organic-related purity (A260/230) ranged from 1.6 to 2.0, with mean values of 1.9 in roe deer and red deer and 2.0 in fallow deer. Neither species (Figure S5a,c) nor tissue origin (Figure S5b,d) nor their interaction had any effect on A260/280 or A260/230 purity ratios.

Genotyping using species-specific multiplex STR systems was successful for all species and sample types using the previously described multiplex kits and PCR settings. Similarly, the multiplex fallow deer-specific system developed in this study, which allows for the simultaneous amplification of eight tetrameric microsatellites, also worked successfully. For each sample, a complete genetic profile could be generated from all extracted DNA isolates (*n* = 60), with genotypes from samples of the same individual being identical and no identical profiles observed between samples from different individuals (Tables S3–S5).

### Case Study

Based on genotyping results obtained with the DNA extraction protocol described in this study and the red deer-specific multiplex STR systems, it was possible to establish that the deer skull and antler shared the same genetic profile (Table S6). That is, the autosomal genetic profiles detected from the samples were identical at the analyzed loci. The likelihood ratio evaluated using the match probability yielded an LR greater than 10^9^ (LR = 1.15 × 10^9^), corresponding to an extremely high probability that the skull and antler originated from the same individual, based on available Hungarian red deer population data [7,26].

## 4. Discussion

Reliable DNA extraction from calcified tissues such as bone, ivory, and antler is essential for wildlife forensics, conservation genetics, food safety, evolutionary biology, and ancient DNA studies [2,3,4,5,6,7,8,9,10,11,12,14,16,17,18,27,28]. While antlers of various deer species have been studied previously (Table S1), protocols for fallow deer antlers and trophies remain underexplored. Traditional methods often require expensive milling equipment, liquid nitrogen, or lengthy demineralization steps and may yield degraded DNA. Over the past few decades, a variety of extraction strategies have been developed, each with distinct advantages and limitations. Phenol–chloroform–isoamyl alcohol extraction results in high yield and is broadly applicable but involves hazardous reagents and labor-intensive handling [29,30]. Silica spin columns provide high-purity DNA with user-friendly workflows, though at a higher cost, whereas magnetic bead-based methods support automation and high-throughput processing but require specialized instrumentation [30,31]. Chelex^®^ extraction is rapid and economical but typically yields DNA of lower purity [32,33]. Selection of a method should therefore balance yield, purity, cost, sample type, and laboratory capacity.

In this study, we optimized a simplified, low-cost protocol for deer antlers using mechanical homogenization (TissueLyser), organic extraction, and Microcon^®^ centrifugal filtration. This approach avoids bone mills and liquid nitrogen while consistently producing high DNA yields, particularly from the antler shaft, which appears better preserved and less inhibitor-rich than the pedicle [4]. Avoiding marrow-rich material likely enhanced DNA quality, as lipids can interfere with extraction and filtration. DNA yield and purity varied among species and sample types, as confirmed by Qubit and Nanodrop measurements. Although the sample size per species was limited (10 individuals), the design allowed preliminary statistical comparison and validation of reproducibility. The protocol proved reproducible, PCR-compatible, and suitable for laboratories with limited resources. Compared with earlier protocols requiring large sample amounts [3,16] or specialized grinding tools [2,4,11,12], our method is minimally destructive yet compatible with downstream applications, including the examination of the nuclear DNA.

Fluorometric quantification (Qubit) provided more accurate estimates of double-stranded DNA than spectrophotometric Nanodrop measurements, which can overestimate concentrations due to co-absorption of proteins, phenol, or other contaminants [34,35,36,37,38,39]. Despite occasional deviations in A_260_/A_280_ and A_260_/A_230_ ratios, PCR amplification and STR genotyping were successful across all species and sample types. Paired comparisons revealed highly similar values for silica column- and phenol-based DNA extracts, typically within the expected range (~1.7–2.0), indicating that the substantial increase in DNA yield was not accompanied by a deterioration in spectrophotometric purity. However, greater variability in purity values was observed for the silica column-based method, likely attributable to measurement bias at low DNA concentrations, where spectrophotometric assessments are known to be less reliable [34].

Identical genotypes in replicate samples confirmed method repeatability, and a case study demonstrated forensic utility by matching a skull and antler from the same individual.

Previous approaches primarily relied on (1) EDTA-based demineralization followed by classical organic extraction and ethanol precipitation, or (2) commercial silica column kits. The former, suitable for degraded or museum-grade samples, requires extended digestion (24–36 h) and efficiently removes inhibitors such as collagen and humic substances [3,4,5,11,13,22]. Silica kits perform well with fresh or less degraded tissue but may underperform with calcified or inhibitor-rich samples unless modified (EDTA, Chelex) [2,7,8,10,16]. Notably, many studies omit details on sample amounts or extraction protocols, and DNA purity is often not reported. Our protocol was thoroughly evaluated: although purity was not ideal in all cases, downstream PCR and genotyping were successful.

Phenol-based methods generally maximize yield, whereas silica columns provide higher purity. Where both parameters are critical, combining phenol extraction with column purification may be optimal. Our results demonstrate that efficient DNA recovery from antlers is feasible without costly equipment. Short enzymatic digestion (4 h) with bead-based homogenization and Microcon^®^ filtration reduces workflow time, minimizes sample loss, and eliminates specialized equipment while producing DNA suitable for high-quality genotyping and downstream applications. Given the toxicity of phenol-based reagents, the protocol should only be carried out in laboratories equipped with appropriate chemical safety infrastructure (e.g., certified fume hoods) in accordance with institutional safety regulations.

## 5. Conclusions

A modified, short, and cost-effective DNA extraction protocol was developed for the pedicle part of prepared skulls and antlers from various deer species. The method does not require a bone mill, yet efficiently isolates high-yield, high-purity DNA even from decades-old antler material. Its novelty lies in demonstrating that the basal antler region is suitable for DNA extraction too and that the protocol performs successfully on fallow deer, a species not previously examined in this context. DNA quality and quantity were consistently sufficient for downstream analyses, as confirmed by spectrophotometric, fluorometric, microsatellite-based genotyping, and case-study validations. By eliminating the need for specialized equipment, this approach broadens access to reliable molecular analyses in laboratories with limited infrastructure and supports a wide range of applications, including wildlife forensics, conservation genetics, and other studies (e.g., EWAS, epigenome-wide association studies) requiring high-yield DNA recovery from calcified tissues.

## Figures and Tables

**Table 1:** DNA concentrations extracted from different parts of the prepared trophy skulls of the three deer species investigated. The “a” or “t” designation in the sample identifier indicates the sampling area within the prepared trophy. a: antler shaft; t: pedicle on the trophy skull; Avg.: average values. The bold formatting indicates: “Average values obtained using the modified phenol-based method.”

	Roe Deer (*Capreolus capreolus*)					Red Deer (*Cervus elaphus*)					Fallow Deer (*Dama dama*)				
		Concentration (ng/µL)		Purity OD			Concentration (ng/µL)		Purity OD			Concentration (ng/µL)		Purity OD	
	Sample	Qubit	Nano-Drop	260/280	260/230	Sample	Qubit	Nano-Drop	260/280	260/230	Sample	Qubit	Nano-Drop	260/280	260/230
Preliminary comparative study	Silica-based method														
	Cc1a	0.3	20.3	1.4	0.8	Ce1a	0.5	16.0	1.8	0.3	Dd1a	0.4	60.8	2.0	1.5
	Cc1t	0.2	39.5	2.0	4.2	Ce1t	1.9	19.5	2.0	0.1	Dd1t	0.4	52.9	1.9	1.8
	Cc2a	0.3	38.3	2.0	13.2	Ce2a	1.6	11.8	1.7	1.1	Dd2a	0.5	57.2	2.0	2.8
	Cc2t	0.1	47.4	2.0	2.6	Ce2t	0.3	38.0	2.0	1.0	Dd2t	0.4	64.6	1.9	1.8
	Cc3a	10.6	34.3	2.0	1.9	Ce3a	0.2	4.0	1.6	0.6	Dd3a	0.4	42.4	2.1	5.5
	Cc3t	0.3	53.4	2.1	2.9	Ce3t	2.3	76.2	1.9	0.5	Dd3t	0.1	55.4	1.8	1.8
	Cc4a	0.3	37.6	2.2	1.3	Ce4a	0.2	42.8	1.9	−57	Dd4a	0.1	47.0	1.9	2.4
	Cc4t	0.6	7.1	1.4	−3.0	Ce4t	0.2	51.4	1.8	3.6	Dd4t	2.3	63.7	1.9	2.5
	Avg.	1.6	34.7	1.9	3.0	Avg.	0.9	32.5	1.8	−6.2	Avg.	0.6	55.5	1.9	2.5
	Modified phenol-based method														
	Cc1a	35.3	38.9	1.9	2.4	Ce1a	17.5	391.0	1.9	1.9	Dd1a	41	340	1.9	2.0
	Cc1t	59.6	117	1.7	1.8	Ce1t	850	485	1.9	2.2	Dd1t	30	552	2.0	2.1
	Cc2a	5.8	1752	1.9	1.6	Ce2a	158	252	1.8	2.0	Dd2a	35.7	337	1.9	2.0
	Cc2t	89.7	134	1.7	1.8	Ce2t	40.0	88.0	1.7	1.8	Dd2t	18.7	337	1.9	2.0
	Cc3a	44.0	1191	1.8	1.8	Ce3a	135	182	1.8	1.9	Dd3a	18.1	243	1.8	2.0
	Cc3t	28.4	655	1.8	1.7	Ce3t	411	353	1.8	1.9	Dd3t	14.2	275	1.8	1.9
	Cc4a	16.6	397	1.9	1.8	Ce4a	57.8	310	1.8	1.9	Dd4a	12.3	149	1.8	2.0
	Cc4t	14.5	169	1.8	2.2	Ce4t	32.8	162	1.8	1.9	Dd4t	17.1	230	1.8	1.9
	Avg.	36.7	557	1.8	1.9	Avg.	213	278	1.8	1.9	Avg.	23.4	308	1.8	2.0
Validation study	Cc5a	15.0	219	1.9	2.2	Ce5a	11.8	342	1.9	2.0	Dd5a	18.4	461	1.9	2.0
	Cc5t	7.3	342	1.9	2.1	Ce5t	448	160	1.7	1.9	Dd5t	6.9	381	1.9	1.9
	Cc6a	33.4	380	1.8	1.8	Ce6a	19.8	101	1.9	1.9	Dd6a	25.8	317	1.9	1.9
	Cc6t	38.0	256	1.8	1.8	Ce6t	59.6	98.0	1.8	2.1	Dd6t	24.6	279	1.9	2.1
	Cc7a	36.5	218	1.8	2.0	Ce7a	64.6	759	1.8	2.3	Dd7a	25.4	269	1.9	2.1
	Cc7t	1.6	52.1	1.7	1.9	Ce7t	66.2	572	1.8	1.7	Dd7t	12.3	415	1.9	2.1
	Cc8a	63.2	1217	1.8	1.7	Ce8a	346	614	2.1	2.2	Dd8a	76.4	361	1.9	2.0
	Cc8t	40.8	333	1.9	2.0	Ce8t	40.0	110	1.8	2.0	Dd8t	22.4	143	1.8	2.2
	Cc9a	152.0	224	1.8	1.9	Ce9a	50.4	112	1.8	2.1	Dd9a	19.5	228	1.8	1.9
	Cc9t	61.8	165	1.8	2.2	Ce9t	98.0	114	1.8	1.7	Dd9t	0.6	156	1.7	1.9
	Cc10a	62.0	579	1.9	1.9	Ce10a	294.0	28.0	1.6	1.7	Dd10a	79.4	120	1.7	1.8
	Cc10t	71.0	3605	2.0	1.8	Ce10t	271.0	379.0	1.8	2.0	Dd10t	21.7	102	1.8	1.8
	Avg.	48.5	632	1.8	1.9	Avg.	147	282	1.8	1.9	Avg.	27.8	269	1.8	2.0
	**Avg.**	**43.8**	**602**	**1.8**	**1.9**	**Avg.**	**174**	**281**	**1.8**	**1.9**	**Avg.**	**26.0**	**285**	**1.8**	**2.0**

## Data Availability

The original contributions presented in this study are included in the article/Supplementary Material. Further inquiries can be directed to the corresponding author.

## Supplementary Materials

The following supporting information can be downloaded at: https://www.mdpi.com/article/10.3390/ani16071056/s1, Table S1: Genetic studies performed on antlers of deer species; Table S2: Details of the 8-plex fallow deer multiplex PCR; Figure S1: Quality assessment for the integrity of DNA isolates by gel electrophoresis; Figure S2: Comparisons of DNA concentrations obtained with silica column- and phenol-based extraction across three cervid species; Figure S3: Paired comparison of A_260_/A_280_ (a) and A_260_/A_230_ (b) purity ratios for DNA extracted with a silica column and the modified phenol-based protocol in three cervid species; Figure S4: Qubit and Nanodrop DNA concentration measurement by sample type and by species; Figure S5: DNA purity by sample type and by species; Table S3: Genetic profiles of the roe deer antlers and trophies investigated; Table S4: Analytical results of fragments detected in red deer antlers and trophies using two tetrameric 5-plex STR systems; Table S5: Analytical results of fragments detected in fallow deer antlers and trophies using a tetrameric 8-plex STR system; Table S6: Analytical results of fragments detected in red deer antlers and trophies in the case study using two tetrameric 5-plex STR systems. References [2,3,4,5,6,7,8,9,10,11,12,14,16,18] are cited in the Supplementary Materials.

## Abbreviations

The following abbreviations are used in this manuscript:

EDTA	Ethylenediaminetetraacetic Acid
GLMM	Generalized Linear Mixed Model
LMM	Linear Mixed Model
LRT	Likelihood Ratio Test
PCR	Polymerase Chain Reaction
RFU	Relative Fluorescence Unit
STR	Short Tandem Repeat
TE buffer	Tris-EDTA buffer
