# Supplementary material for: A Simplified and Efficient Protocol for DNA Isolation from Deer Antlers and Prepared Trophy Skulls
Source: Animals (Basel). 2026 Mar 30;16(7):1056. doi: 10.3390/ani16071056 (PMC13072065; doi:10.3390/ani16071056)
Supplement: Supplementary file 1 [file animals-16-01056-s001.zip › animals-4129331-supplementary.pdf]

Table S1. Genetic studies performed on antlers of deer species, indicating the purpose of the study, the extraction method of genetic material, the results regarding the quantity and quality of the extracted DNA, and the subsequent molecular methods used. nd: no data available, ON: overnight, h: hour.

**Species**	**Sample type**	**Study aim**	**Sample (g)**	**Grinder /liquid N2**	**Decalcification time, extraction, and purification method**			**DNA conc.**	**OD260/ 280**	**Subsequent analysis**	**Ref.**
Coues' white-tailed deer (*Odocoileus virginianus couesi*)	old antlers (1960-1996)	population genetics	nd	no/no	1h	phenol	ethanol-precipit.	10-50 µg		microsatellite marker amplification	[5]
giant deer (*Megaloceros giganteus*)	bone, antler from museum	identification	1	no/no	36 h	phenol-chloroform	2-propanol precipit.,	nd	nd	mitochondrial gene sequencing	[3]
Coues' white-tailed and mule deer *(Odocoileus virginianus couesi* and *O. hemionus)*	weathered antler	DNA stability	nd	no/no	nd	nd	nd	nd	nd	microsatellite marker amplification	[6]
red deer *(Cervus elaphus)*	middle-aged, old antlers	ecological	0.2	yes/yes	24 h	phenol-chloroform-isoamylalcohol	ethanol precipit.	50-100 ng/µL	nd	microsatellite marker amplification	[4]
Hungarian red deer *(Cervus elaphus hippelaphus)*	shed antler	forensic case	nd	nd	ON	dextran blue	ethanol precipit.	nd	nd	microsatellite marker amplification	[7]
red deer (*Cervus elaphus*)	antler velvet	species identification	0.05	yes/no	ON	Tris-EDTA-saturated phenol, and silica membrane-based DNA extraction	ethanol-precipitation	27 - 34.5 ng/µL	nd	mitochondrial gene sequencing	[11]
sika deer (*Cervus nippon*)	antler velvet tip	transcripto-mics	nd	no/yes	nd	phenol-chloroform and TRIzol™ Reagent	ethanol precipit.		nd	transcriptome and microRNA sequencing	[9]
taruca and huemul (*Hippocamelus antisensis* and *H. bisulcus*)	shed antler connective tissue	non-invasive DNA isolation	10	no/no	nd	1) Wizard Genomic DNA kit 2) phenol-chloroform 3) SDS+ Prot. K+NaCl precip.	1-2) isopropanol precipit.	0.9-13.4 ng/µL	nd	mitochondrial gene sequencing	[16]
reindeer (*Rangifer tarandus*)	antler mesenchyme	transcripto-mics	nd	no/no	nd	TRIzol™ Reagent	RNA purification		nd	transcriptome sequencing	[14]
sika deer (*Cervus nippon*)	antler tip	DNA methylation	nd	no/no	nd	silica membrane-based DNA extraction system		nd	nd	fluorescence-labelled methylation-sensitive amplified polymorphism	[10]
sika deer (*Cervus nippon hortulorum*)	antler tip	DNA methylation	nd	no/no	nd	silica membrane-based DNA extraction system		> 200 ng/µL	1.8-2.0	bisulfite sequencing, quantitative real-time PCR	[8]
Roe deer (*Capreolus capreolus*)	antler	population and landscape genetics	0.2	yes/yes	nd	phenol-chloroform		nd	nd	microsatellite marker amplification	[18]
sika and red deer (*Cervus nippon* and *Cervus elaphus*)	antler	species identification	0.1	yes/yes	6 h	silica membrane-based DNA extraction system		573-1272 ng/µL	1.8-2.0	mitochondrial gene sequencing	[12]
red deer and moose (*Cervus elaphus* and *Alces alces*)	ancient antler	species identification	0.05	yes/no	ON	modified silica-membrane base DNA extraction	ethanol precipit.	nd	nd	mitochondrial genome sequencing	[2]

Table S2. Details of the 8-plex fallow deer multiplex PCR: primer sequences for eight autosomal STRs, size range, and primer concentrations (equal amounts for forward and reverse primers).

Marker	Size range (bp)	Forward Primer	Reverse Primer	Dye	Primer concentration
**ApoV146**	143–148	GGGCCCTCAATTCTCTTCC	GGAGACATCACATTCCCTGAC	VIC	200 nM
**Mgoua20**	193–197	ACAACTGGAGAAAACCCTTGTG	AGCCTTTAGAGATGTTCTGTTTGG	PET	300 nM
**OheF**	199–211	CAGGCGATCAAGAAATGTGG	GTGGCTTCTGGATGGAGAAC	VIC	100 nM
**T268**	224–240	ATTCCCTTCTCCAGTGTATG	GATGATAACAGCTCAACAGATC	6-FAM	400 nM
**OheQ**	248–264	AATGTGTCAGTGAAGGTCTTC	ATCCAGGCAACCATCTAG	VIC	450 nM
**T107**	283–308	ACATCCGTTCAGGTGTGA	CCAGAGGTAAGATAAATGGTGA	6-FAM	150 nM
**C32**	285–289	ACAACTGTGTGAGCCAATAC	AGCAAGTGAAGAAGAATGTTC	PET	200 nM
**276**	376–380	AAACAGAACATTCACCAGAAA	CTCCCAGACACACAGAACAA	VIC	450 nM

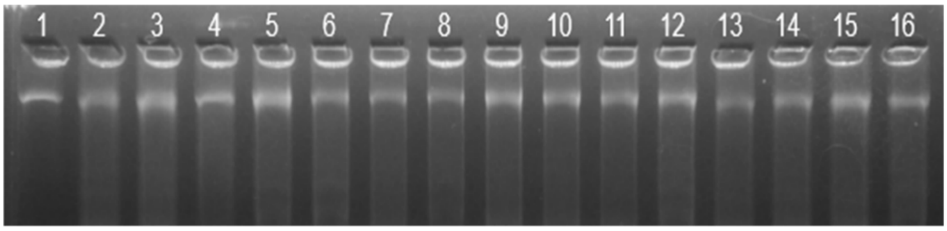

Figure S1. Quality assessment for the integrity of DNA isolates (n = 16) by gel electrophoresis using 1% agarose gel

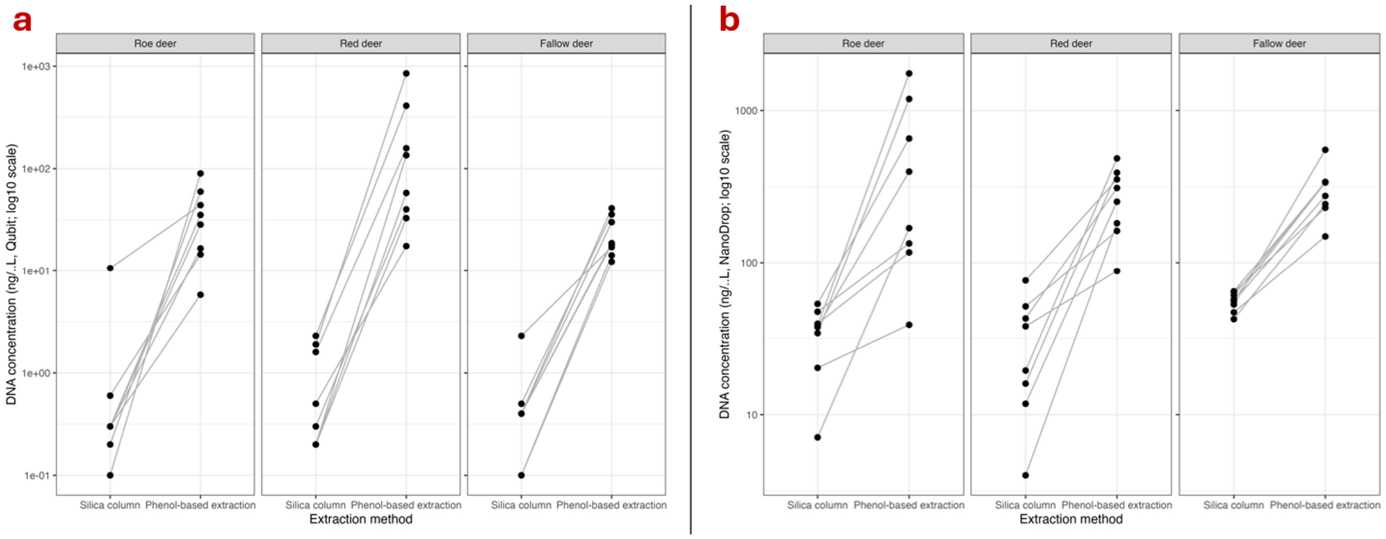

Figure S2. Comparisons of DNA concentrations obtained with silica column and a phenol-based extraction across three cervid species (n = 8 paired samples per species). Each line connects paired silica-column measurements from the same individual sample. The y-axis is shown on a $\log_{10}$ scale to visualise both low and high concentration values. The fold-increase ranged from approximately 23-fold in roe deer to 40-fold in fallow deer and exceeded 200-fold in red deer using the Qubit measurement (a), and Nanodrop-based DNA concentrations showed the same trend, with 5.5- to 16.0-fold higher mean values for the phenol-based protocol across species (b).

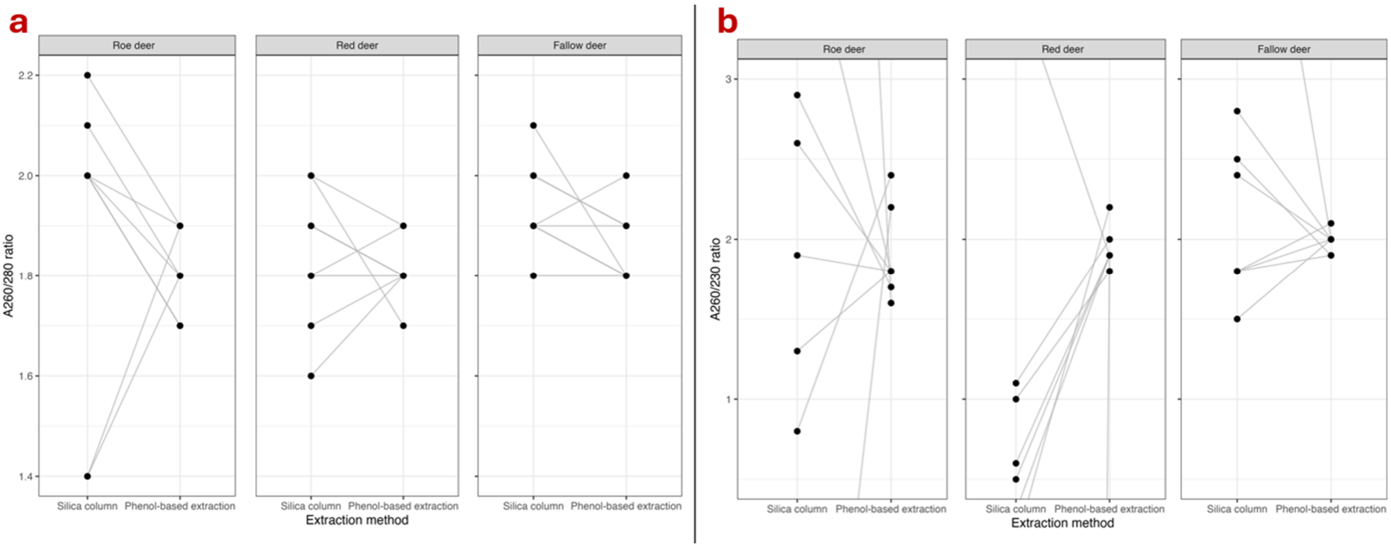

Figure S3. Paired comparison of A_{260}/A_{280} (a) and A_{260}/A_{230} (b) purity ratios for DNA extracted with silica column and the modified phenol-based protocol in three cervid species (*n* = 8 paired samples per species). Each pair of points represents measurements from the same individual sample. A_{260}/A_{280} and A_{260}/A_{230} values were highly similar between extraction methods within each species, and Wilcoxon signed-rank tests did not detect significant differences (all $p > 0.05$). The phenol protocol yielded slightly more consistent distributions, whereas Qiagen extracts displayed somewhat wider variability in roe deer and fallow deer. No systematic method-specific pattern was observed, in agreement with the paired analyses.

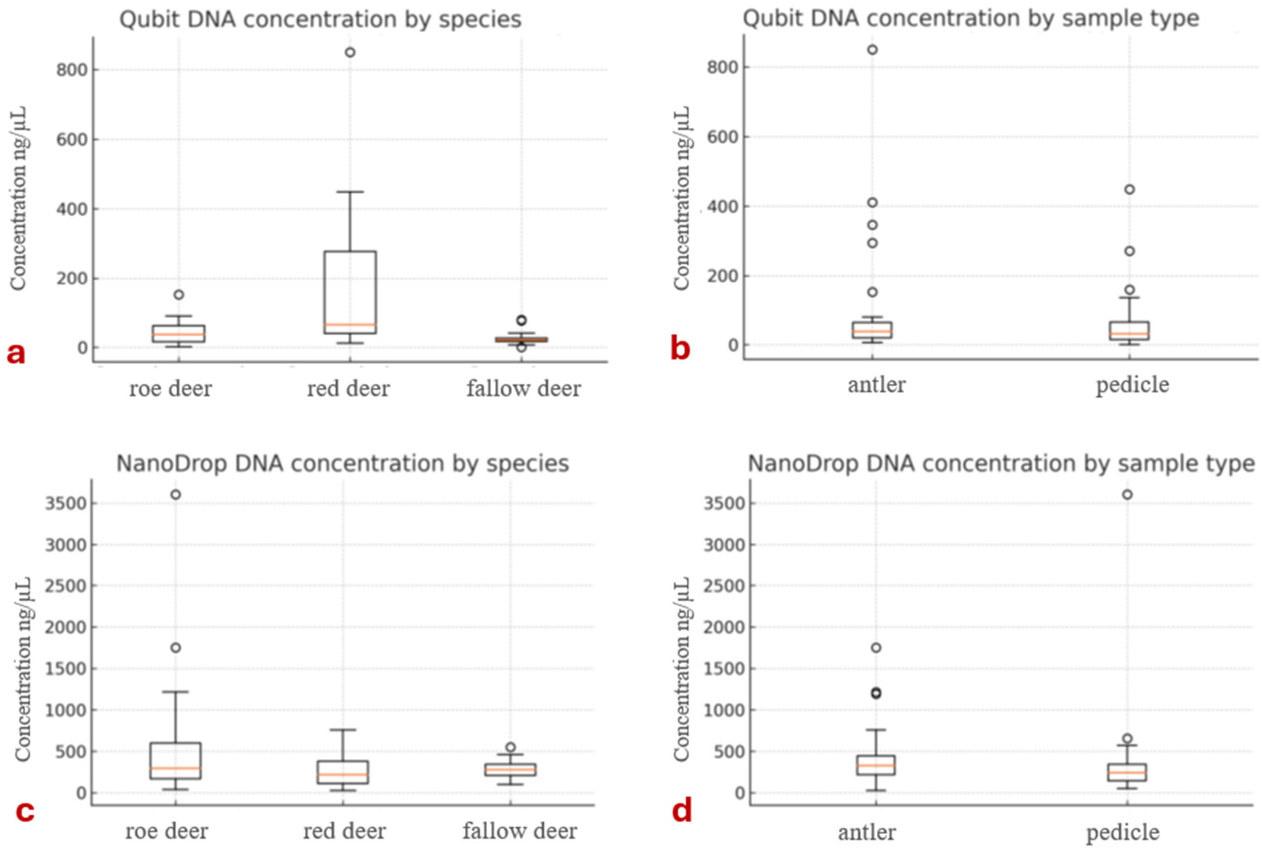

Figure S4. Qubit and Nanodrop DNA concentration measurement by sample type and by species. Boxes indicate the interquartile range (IQR), horizontal lines show medians, whiskers extend to 1.5 × IQR, and points represent individual measurements. DNA concentration measured with Qubit was significantly influenced by species (a); red deer samples had significantly higher DNA concentrations than both fallow deer and roe deer. Tissue origin and its interaction with species did not affect Qubit-based concentration values (b). For Nanodrop measurements, neither species nor its interaction with tissue origin influenced DNA concentration (c–d). However, tissue origin itself had a significant effect, with antler samples yielding higher concentrations than pedicle samples.

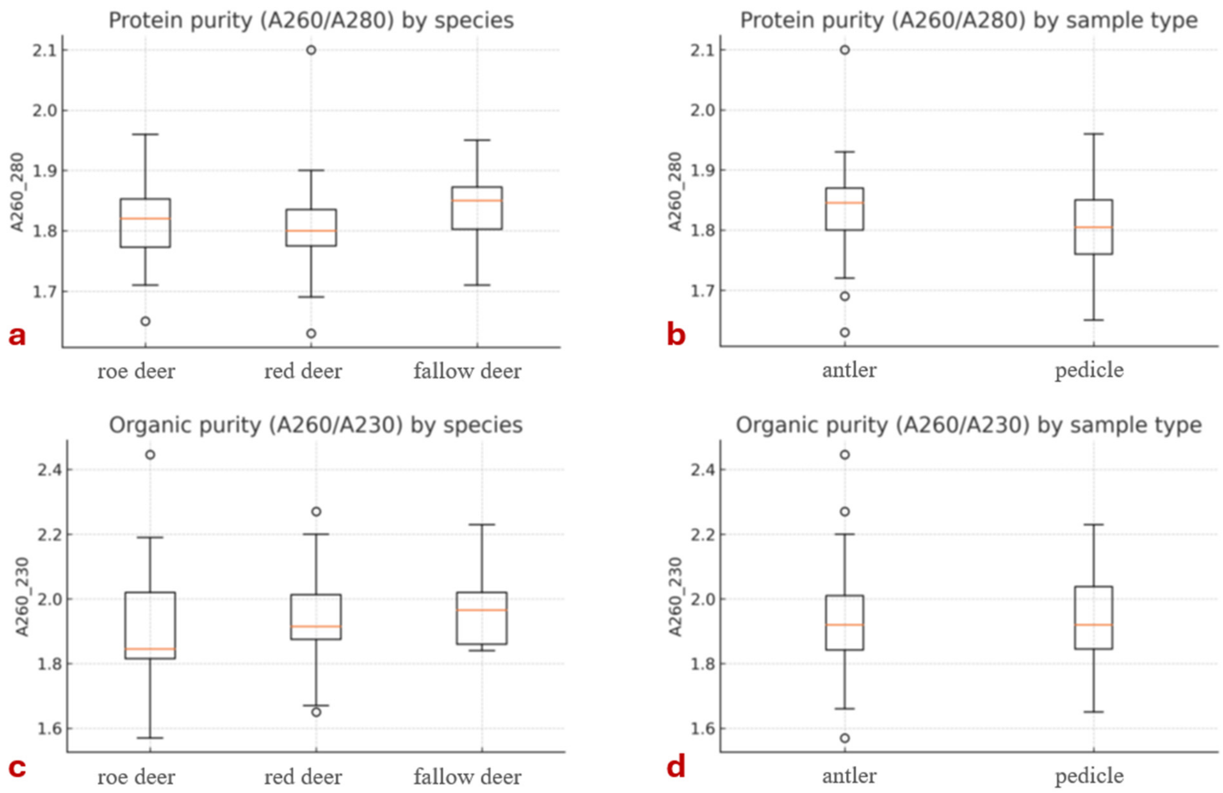

Figure S5. DNA purity by sample type and by species. Boxes and whiskers as in Figure 3. Neither the species (a,c), tissue origin (b,d), nor their interaction affected A260/280 and A260/230 purity ratios (260/280: null model had higher logLik values with all $p < 0.065$; A260/230 ratio: all $p > 0.584$). No correlation was observable between Qubit and Nanodrop DNA concentration values ($\rho = -0.06$, df = 56, $p = 0.680$).

Table S3: Genetic profiles of the roe deer antlers and trophies investigated, based on the size of the detected fragments located in the allele fragment size-interval of the roe deer-specific STR markers. Bold: sample. Bold-italics: examined loci, a: antler, t: trophy skull, Cc: *Capreolus capreolus*,

	Capcap2		***Capcap17***		***Capcap10***		***Capcap36***		***Capcap3.1***		***Capcap25***		***Capcap31***		***Capcap35***		***Capcap1***		***Capcap15***		***Capcap29***		***Capcap5***		***Y***	***X***
**Cc1a/t**	11	12	10	11	11	11	14	15	10	11	10	10	10	11	9	9	10	11	7	7	8	12	10	14	Y	X
**Cc2a/t**	11	11	11	11	11	12	14	15	9	10	10	10	9	9	12	12	7	10	6	7	10	12	12	14	Y	X
**Cc3a/t**	12	13	11	11	11	13	13	14	10	10	10	10	10	11	11	12	9	11	7	7	8	12	9	12	Y	X
**Cc4a/t**	11	14	10	11	7	13	13	13	9	12	10	10	10	11	7	9	7	10	6	8	6	11	12	12	Y	X
**Cc5a/t**	12	14	10	11	7	14	12	16	9	11	9	10	12	12	7	7	7	10	7	7	5	8	12	14	Y	X
**Cc6a/t**	13	15	10	11	11	13	14	16	9	10	10	10	11	12	9	9	7	10	7	9	8	12	9	9	Y	X
**Cc7a/t**	12	15	10	11	11	12	12	17	9	9	10	11	11	11	8	9	10	11	7	7	8	11	9	9	Y	X
**Cc8a/t**	13	13	10	11	11	14	11	14	9	10	8	10	10	11	9	12	10	11	7	9	8	8	12	14	Y	X
**Cc9a/t**	13	13	11	11	11	14	12	14	6	10	6	10	10	11	11	11	7	10	6	7	10	11	11	12	Y	X
**Cc10a/t**	14	15	10	14	11	11	13	13	9	11	9	11	9	12	9	9	10	10	7	9	8	12	9	13	Y	X

Table S4: Analytical results of fragments detected in red deer antlers and trophies using two tetrameric 5-plex STR systems, within the allele size range of red deer-specific STR markers (the nucleotide lengths of registered alleles are relative sizes, based on the applied size standard). Bold: sample. Bold-italics: examined loci, a: antler, t: trophy skull, Ce: *Cervus elaphus.*

	DeerPlex I									
	C229		***T16***		***T193***		***T108***		***T123***	
**Ce1a/t**	110.4		175.2	200.4	149.5	157.7	132.7	150.1	116.8	124.7
**Ce2a/t**	110.5	114.5	188.4		153.6	165.9	120.5	124.6	117.1	137.1
**Ce3a/t**	110.6	118.5	145.0	188.5	153.7	169.8	120.6		120.8	
**Ce4a/t**	114.5		175.3	203.4	145.1	153.7	124.6	145.8	112.9	132.8
**Ce5a/t**	110.5		164.0	183.3	181.7		128.5	132.6	81.3	105.4
**Ce7a/t**	110.4	114.4	146.2	188.3	169.7	181.7	124.6	128.6	81.3	128.8
**Ce8a/t**	110.5		183.3	188.4	136.2	177.8	120.5	132.7	105.4	
**Ce9a/t**	102.7	114.5	183.3	188.4	169.8	177.7	128.6	132.7	117.1	
**Ce10a/t**	110.6	114.5	179.3	192.4	128.1	157.8	120.6	132.7	81.3	117.0

	DeerPlex II									
	T501		***T172***		***C01***		***T156***		***T507***	
**Ce1a/t**	151.5	162.8	107.7	119.7	220.4	255.5	173.4		195.4	203.2
**Ce2a/t**	151.4	152.4	103.7	145.0	212.5		131.7	165.5	191.5	214.9
**Ce3a/t**	143.4	144.5	103.8	115.7	224.3	243.9	131.8	163.3	191.4	214.9
**Ce4a/t**	152.4	161.6	132.0		212.5	232.1	157.4	181.4	195.4	
**Ce5a/t**	159.7	162.8	103.8	132.0	212.4	243.8	131.9	165.5	183.6	195.4
**Ce7a/t**	161.6		140.7	145.1	208.7	216.4	149.2	157.4	187.6	195.4
**Ce8a/t**	139.0	182.0	111.6	149.7	212.6	243.9	163.3		183.6	191.4
**Ce9a/t**	144.6	161.6	132.0	145.0	212.5	243.8	140.1		195.4	214.8
**Ce10a/t**	152.4	161.5	103.7	153.8	208.7	232.0	140.1	177.3	187.4	210.9

Table S5: Analytical results of fragments detected in fallow deer antlers and trophies using a tetrameric 8-plex STR system, within the allele size range of fallow deer-specific STR markers (the nucleotide lengths of registered alleles are relative sizes, based on the applied size standard.) Bold: sample. Bold-italics: examined loci, a: antler, t: trophy skull, Dd: *Dama dama*.

	T268	***T107***	***ApoV146***	***OheF***	***OheQ***	***C276***	***Mgoua20***	***C32***
**Dd1a/t**	209.4	264.4	122.4 126.7	183.8	229.8	362.0	180.0	276.6
**Dd2a/t**	209.3	264.5	122.3	183.7	229.7	362.0 365.8	180.1 184.1	276.7
**Dd3a/t**	209.3	264.5	122.3	183.7	229.7	361.9	180.3	276.7
**Dd4a/t**	209.3 225.1	264.5 268.5	122.4 126.6	183.9	229.8	361.9 365.7	180.4	276.7
**Dd5a/t**	209.3	264.4	122.4 126.6	183.7	229.8	362.0	180.2	276.7
**Dd6a/t**	209.4	264.5	122.5 126.8	183.8 196.1	229.8	361.9 365.7	180.4	276.7
**Dd7a/t**	209.4	284.3	122.4	183.8	229.8	362.0 365.9	180.3	276.7
**Dd8a/t**	209.3	264.5	122.4	183.8	229.8	362.0	180.2 184.2	276.7
**Dd9a/t**	209.3	264.5	122.3	183.8	229.8	361.9 365.7	180.0	276.7
**Dd10a/t**	209.3	264.5 284.3	122.4 126.6	183.8. 196.0	229.8	362.0 365.8	180.3	276.7

Table S6: Analytical results of fragments detected in red deer antlers and trophies in the case study using two tetrameric 5-plex STR systems, within the allele size range of red deer-specific STR markers (the nucleotide lengths of registered alleles are relative sizes, based on the applied size standard). Bold: sample. Bold-italics: examined loci.

	DeerPlex I				
	C229	***T26***	***T193***	***T108***	***T123***
**1/1. trophy skull**	110.6 114.5	159.0 183.4	140.5	120.4 141.2	117.0 120.8
**2/1. antler**	110.5 114.5	159.0 183.3	140.5	120.5 141.4	116.9 120.7

	DeerPlex II				
	T501	***T172***	***T156***	***C01***	***T507***
**1/1. trophy skull**	149.2 162.8	127.8 149.6	157.4 189.6	224.3 243.8	183.6 211.0
**2/1. antler**	149.2 162.8	127.9 149.6	157.4 189.6	224.3 243.8	183.6 211.0
